# Health status risk factors and quality of life in 75–84-year-old individuals assessed for dementia using the short 10/66 dementia diagnostic schedule

**DOI:** 10.7717/peerj.12040

**Published:** 2021-08-19

**Authors:** Gershwin Davis, Nelleen Baboolal, Vrijesh Tripathi, Robert Stewart

**Affiliations:** 1 Department of Paraclinical Sciences, Faculty of Medical Sciences, The University of the West Indies, St. Augustine, St. Augustine Campus, Trinidad, Trinidad and Tobago; 2 Department of Clinical Medical Sciences, Faculty of Medical Sciences, The University of the West Indies, St. Augustine, Trinidad, Trinidad and Tobago; 3 Department of Mathematics and Statistics, Faculty of Science and Technology, The University of the West Indies, St. Augustine, Trinidad, Trinidad and Tobago; 4 Institute of Psychiatry, Psychology and Neuroscience, King’s College London,, London, United Kingdom; 5 South London and Maudsley NHS Foundation Trust, London, United Kingdom

**Keywords:** Dementia, Risk factors, Short 10/66 diagnostic schedule

## Abstract

**Background:**

Trinidad is an island that not only has a population at high vascular risk but also one that is in epidemiological transition with high dementia prevalence. The aim of the study was to investigate modifiable risk factors associated with dementia in middle-old (75–84 years) individuals.

**Methods:**

As part of a large national community survey of dementia prevalence in Trinidad, 811 people aged 75–84 years were evaluated for dementia using the 10/66 short algorithm. Demographic data collected included information on age, gender, ethnicity, religion, education, occupation, living accommodation, smoking, alcohol consumption, self-reported medical conditions, impairments and ability to do instrumental activities of daily living (IADL). Data were analysed using multivariable logistic regression models.

**Results:**

Of the 811 participants, nearly 55% were female. The mean age was 78.8 (SD = 2.8) years and dementia was present in 198 (24.4%). Having less than ten years of education, being an agricultural worker, skilled labourer or housewife and having more than four co-morbidities were significantly associated with dementia. The odds ratio for dementia for those having self-reported stroke was 4.93 (95% CI [2.64–9.23]) and for diabetes was 1.76 (95% CI [1.17–2.65]) adjusting for age, age^2^, gender, ethnicity, religion, education and occupation. Impairment in eyesight, hearing, climbing stairs, and walking were also more common in the group with dementia. Ability to perform IADLs was linked with dementia. Individuals with dementia were more likely to be unable to perform any of the eight IADLs. Those who did not exercise at all (OR 6.95, 95% CI [2.02–23.90]) and those who did low exercise (OR 1.83, 95% CI [1.07–3.13]) compared to those who did moderate to high exercise were also more likely to have dementia.

**Conclusion:**

In the middle-old population in Trinidad having diabetes and stroke, low IADL score, and no exercise were more common in people with dementia.

## Introduction

A dramatic demographic change is occurring world-wide. With increased life expectancy, the oldest segments of the population are increasing at a fast rate. This has given rise to an increase in the number of older individuals with chronic diseases and dementia. Researchers have subdivided old age into various groups (Forman et al., 1992; Zizza, Ellison & Wernette, 2009; Pirkl & Transgenerational Design Matters, 2009). One such division demarcates young-old as persons 65 to 74 years old, middle-old as persons 75 to 84 years old, and oldest-old as persons 85 years old and older. Reed, Lowrey & Vallis (2006) reported that middle-old and oldest-old retirement dwelling adults responded differently to locomotor challenges in cluttered environments. In another study, large differences in demographics and clinical courses were found among cohorts of the youngest-old, middle-old, and oldest-old patients who visited the emergency department (Lee et al., 2018). Differences in health were also notable across these cohorts, and post-hoc tests show significant differences between all groups for instrumental activities of daily living (IADLs) (Norstrand & Chan, 2014).

Trinidad and Tobago is in an advanced stage of demographic transition: the number of children below the age of 15 years has decreased while the number of persons 60 years and older has doubled (Ministry of Health Trinidad, Tobago (MOHTT), 2011). We have recently reported a relatively high prevalence of dementia in the elderly population over 70 years old in Trinidad and Tobago (Davis et al., 2018a; Davis et al., 2018b), a country with an ageing population with high vascular risk (Ministry of Health Trinidad, Tobago (MOHTT), 2012). When our results were compared with a meta-analyses for the Americas (Prince et al., 2013), the prevalence of dementia observed for Trinidad in the 75–79 year age group was 23.5% compared to 8.4% in Latin America and 6.3% in the USA. In the 80–84 age group, the prevalence of dementia was 25.8% in Trinidad compared to 15.4% in Latin America and 11.9% in USA. In those aged 90 years and over, the prevalence of dementia in Trinidad was 57.7% compared to 63.9% reported for Latin America and 47.5% for the USA. To the best of our knowledge, this is the first study to focus upon prevalence and risk factors of dementia in the middle-old population of Trinidad. The use of the short 10/66 dementia diagnostic schedule made the study economically and logistically possible (Davis et al., 2018a; Davis et al., 2018b). This population was notably born before Trinidad and Tobago’s independence, between 1936 and 1945, and had limited opportunities for education and employment. This was a time in Trinidad when educational opportunities were fewer and most individuals were not able to either go to school or stay in the schooling system for long (Ramsaran, 2001). Often boys and girls were married and pushed into employment at an early age.

Older age remains the strongest risk factor for dementia even though it has not been proved that dementia is an inevitable consequence of ageing (Qiu & Fratiglioni, 2018). Many researchers have hypothesized about the complex combination of socioeconomic and cultural factors associated with dementia, although causal pathways have yet to be conclusively demonstrated (Chen & Zissimopoulosa, 2018). Chief factors among these are racial, ethnic and gender differences that are responsible for disparities in opportunities for education and employment. The 2020 report of the Lancet Commission further details risk factors in early life (education), midlife (hypertension, obesity, hearing loss, traumatic brain injury, and alcohol misuse) and later life (smoking, depression, physical inactivity, social isolation, diabetes, and air pollution) as contributors to increased risk of dementia (Livingston et al., 2020).

Ageing is also associated with an increase in the frequency of chronic (non-communicable) diseases as well as an increase in limitations to IADLs and prevalence of a range of disabilities. Notable in Trinidad are chronic disease conditions such as hypertension and diabetes which may be modifiable risk factors for dementia in mid- and late life. These conditions in middle-aged and older adults have been reported to impact social participation and ability to function. Smoking and lack of physical activity have also been shown to affect survival after age 75 (Rizzuto et al., 2012) and cessation of smoking reduces the risk of dementia (Livingston et al., 2020). Poor cardiovascular health including diabetes, obesity, smoking, hypertension and high cholesterol (Chen & Zissimopoulosa, 2018) have been associated with dementia and these conditions have been associated with multimorbidity in Trinidad (La Foucade et al., 2020).

We hypothesize that modifiable factors such as chronic disease conditions, physical impairments and inactivity in the middle-old population are associated with higher occurrence of dementia. We investigated individuals with/without dementia in the middle-old population (75–84 years) with respect to multiple morbidity, impairments, ability to perform IADLs and levels of exercise. In our analysis, non-modifiable factors included were age, gender, ethnicity, religion, as also highest level of educational attainment and occupation. The aim of the study was to identify modifiable risk factors for dementia so that quality of life may be improved for those in advanced years of life.

## Method

A survey was carried out in two phases using a nationally representative sample (CSO, 2011) of the population of Trinidad. The aim was to enrol all persons aged 70 years and over; the details have been reported elsewhere (Davis et al., 2018a; Davis et al., 2018b). This study considers the middle-old (75–84 year) subset of that study population and involves all residents within the age group, living in the randomly selected electoral enumeration districts (EDs) within municipalities in Trinidad. A municipality is defined as a primarily urban political unit, having corporate status and powers of self-government, and an ED is a geographical area comprising approximately 150–200 households; 120 EDs were selected using stratified random sampling with proportional allocation to each municipality stratum, meaning that the number of EDs selected within a municipality was proportional to the population size of the particular municipality. Sample size calculations for the original survey indicated that this would be sufficient to recruit an overall sample of 2000, which would allow estimation of a typical dementia prevalence of 4.5% with a desired precision of ±0.9% at the 95% confidence level using the Wald method.

In phase I, household enumeration was conducted in the 120 randomly selected EDs, with maps provided by the Central Statistical Office that indicated the location of households. In each ED, all households were approached. Field workers went door to door and inquired about individuals living in the household who were ≥70 years. In phase II, field workers revisited the specific households; that is, the households with at least one resident aged ≥70 years, as identified in phase I (door knocking phase), to interview index participants, informants (someone who knew a participant well) and heads of households. All enumerated individuals resident in the 120 EDs and aged ≥70 years were invited to participate. As stated, the report presented here focuses in detail on the middle-old participants aged between 75–84 years old. Ethical approval was granted by the chairman of the ethics committee of The University of the West Indies.

### Recruitment

**Figure 1:**
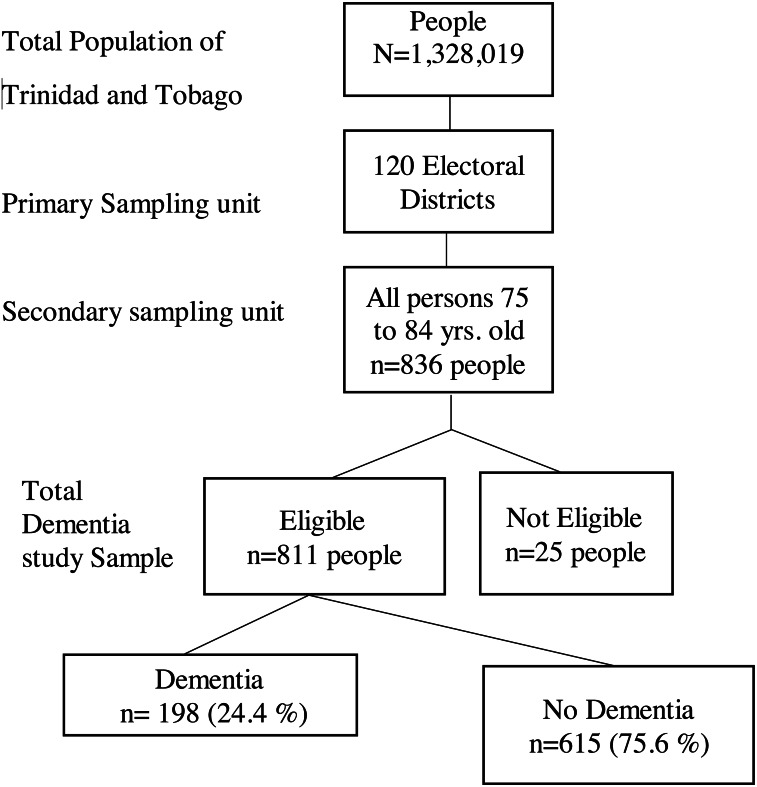
Flow diagram of multistage sampling and response rates.

The study used the validated 10/66 door-to-door interview protocols. Of the survey responders, we identified all individuals in our database (836 persons) who were considered middle-old in the selected electoral enumeration districts (ED). Diagnosis of dementia was derived from the Community Screening Instrument for Dementia (CSI-D; Hall et al., 1993; Hall et al., 2000) administered to participants and informants, the CERAD 10-word list learning task (Morris et al., 1989) and the EURO-D depression screen (Castro-Costa et al., 2008). Output from these instruments was then used to assign dementia status according to the short 10/66 algorithm (Stewart, Guerchet & Prince, 2016) which draws on the output of these instruments and applies a series of regression coefficients to assign a probabilistic diagnosis of dementia (Prince et al., 2003). In the short schedule, the Geriatric Mental State interview is replaced by the EURO-D scale. The algorithm produced output in 811 participants (97%), while the remaining 25 had missing source data that precluded algorithm output. A summary of the sampling methodology is shown in the flowchart below. Figure 1.

### Measurements

In addition to their age and gender, participants were asked to report on their ethnicity, marital status, highest level of education, religion, occupation, type and tenure of accommodation, and living arrangements (See File S1). As part of the survey an informant questionnaire was also done. Ethnicity was recorded according to the most common groups present within the island, including African, Caucasian, Chinese, East Indian, Mixed African and Indian, Indigenous, Mixed Other, Portuguese, Syrian/Lebanese, and others. These were re-coded for analyses as African, East Indian, Mixed African and East Indian, and Mixed other/Others. Religion was recorded as 15 separate denominations including categories for “None” and “Not Stated.” These were then coded as Christian, Hindu, Muslim and Others and “Not Stated” as a group comprising other religions for analyses. Marital Status was categorised as married/co-habiting, divorced/legally separated, never married and widowed. Education was coded as none, primary (*i.e.,* formal education ages 5 to 11 years), secondary (*i.e.,* formal education from 12 to 19 years), or tertiary and other (*i.e.,* education at the university or polytechnic level). Reported previous occupation was categorised as agricultural worker, associate professional, clerical worker/secretary, housewife, manager/administrator, professional, semi-skilled labourer, skilled labourer or unskilled labourer. Previous occupation referred to the major occupation held during working years. These categories were then merged for analyses as associates/professionals/managers, agricultural workers, unskilled or semi-skilled labourers, skilled labourers, and housewives. Type of accommodation were categorised as detached house, and condominium, townhouse, or other. Tenure of accommodation was categorised as privately owned (including by spouse or family), and rented (including from private landlord, National Housing Authority (NHA), housing), or other. Living arrangements of chief respondent were categorised as self (*i.e.,* living alone), with spouse, with spouse and children, with children alone, and with other.

Questions were also asked about the frequency of smoking and alcohol consumption. Participants were asked if they had any of the following 15 medical conditions listed in their lifetime and which persisted in the last 3 months: angina, Alzheimer’s disease, arthritis, asthma, cancer, dementia, depression, diabetes, epilepsy, head injury, heart disease, high cholesterol, hypertension, Parkinson’s disease and stroke. These were then categorised as presence of number of co-morbidities in a person which were coded as none, 1, 2, 3 and greater than/equal to 4. Self-reported medical conditions were recorded as yes/no for arthritis, angina, diabetes, heart disease, high cholesterol, hypertension/BP medicine and stroke in the analyses. The following impairments were also recorded as binary responses: eyesight, hearing, climbing stairs, walking, and ability to speak and understand. An IADL score was generated based upon binary yes/no responses on ability to perform the following tasks: using the telephone, shopping independently, preparing food independently, doing housekeeping, doing laundry, commuting independently, handling finance independently and taking own medicine (Lawton & Brody, 1969). The IADL score ranged from 8 (able to perform all the activities) to 0 (cannot perform any of the activities) (Juva, Makela et al., 1997). Participants were also asked if they considered themselves to be physically active. Physical activity being defined as any bodily movement that is produced by the contraction of skeletal muscle and that substantially increases energy expenditure (CDC, 2017). The responses were recorded as not at all, not very much, fairly, and very much. These were re-coded as no exercise, low exercise, and moderate to high level of exercise for analyses.

### Statistical analyses

Prevalence of dementia was calculated and reported. Univariable and multivariable analyses of factors associated with dementia were carried out using logistic regression on the unweighted sample. To address the non-independence of EDs selected, robust clustered standard errors were used. All analyses are reported in tables with odds ratio (OR), 95% confidence intervals (95% CI), robust standard errors (SE) and *p*-values. The level of significance used was two-sided *p* < 0.05. Stata 14.2 (StataCorp, 2015) was used for all analyses.

We applied univariable logistic regression to model the association between dementia and self-reported medical conditions and quality of life variables. A series of models were developed using logistic regression methods. The models were assessed for calibration and discrimination using area under the Receiver Operating Characteristic (AUROC), goodness-of-fit (GOF) and Hosmer and Lemeshow’s tests. Calibration is a measure of how well predicted probabilities agree with actual observed risks while discrimination is a measure of how well the model can separate those who do and do not have the disease of interest (Cook, 2007). The larger the AUROC curve and the closer to 1, the better the diagnostic effect of the model (Kleinbaum & Klein, 2010). We fitted multivariable logistic regression on self-reported medical conditions related to vascular diseases, arthritis and diabetes (Model I). To address non-linear associations, age squared was added to the model. Age is a known risk factor for dementia, while gender, ethnicity and religion may be confounders for angina, diabetes, heart disease, high cholesterol, hypertension and stroke patients since the incidence, time and length of disease may be effected by hormone levels, ethnic make-up and dietary practices. Hence, the model was adjusted for age, age^2^, gender, ethnicity and religion. Education and occupation were then added to the model to account for differences in lifestyles arising out of educational attainments and practising occupation for the majority of their lives in Model II. In the third model, we included age, age^2^, gender, ethnicity, religion, education, occupation, marital status, living arrangements, type of accommodation, smoking and alcohol consumption to assess their association with dementia (Model III). These additional factors were added to account for their loneliness, ability to look after themselves, avoid smoking and limit their alcohol consumption to acceptable levels. A similar approach was taken for developing multivariable models for quality of life variables (impairments, IADLs and exercise) adjusting for age, age^2^, gender, ethnicity and religion (Model IV), adjusting for age, age^2^, gender, ethnicity, religion, education and occupation (Model V), and adjusting for age, age^2^, gender, ethnicity, religion, education, occupation, marital status, living arrangements, type of accommodation, smoking and alcohol consumption (Model VI). The best models were chosen based model specification tests and performance.

## Results

There were 836 participants aged 75–84 in the analyzed sample. The mean age was 78.8 (SD 2.8) years. Dementia was assessed in 811 participants and was found to be present in 198 (24.4%), of whom 88 were males (dementia prevalence 24.0%) and 110 were females (dementia prevalence 24.7%). Sample characteristics and dementia prevalence by group are described in Table 1. Prevalence of dementia was highest among those who were of East Indian ethnic descent, followers of Hindu faith, those who had not received formal education, agricultural workers and housewives, those living in detached houses, those who lived with children alone, having presence of four or more co-morbidities, those with stroke, those who never drank alcohol, and those who did not exercise at all.

**Table 1:** Background characteristics, prevalence and univariable analyses of socio-demographic, self-reported medical and quality of life covariates associated with dementia.

Covariates	Total	Prevalence of dementia (95% CI)	OR (95% CI)	*p*-value
**Age (yrs) Mean (SD)**	78.8 (2.8)		1.0580 (0.999, 1.121)	0.055
**Age^**2**^**			1.0004 (0.999, 1.001)	0.053
**Gender N(%)**				
Male	366(45.13)	24.04(19.92, 28.71)	1	
Female	445(54.87)	24.72(20.92, 28.96)	1.04(0.75, 1.43)	0.824
**Ethnicity N(%)**				
African	310(38.27)	23.55(19.13, 28.62)	1	
East Indian	303(37.41)	28.71(23.87, 34.09)	1.31(0.91, 1.88)	0.146
Mixed African & East Indian	36(4.44)	19.44(09.21, 36.49)	0.78(0.33, 1.86)	0.581
Mixed other/others	161(19.88)	19.25(13.82, 26.16)	0.77(0.48, 1.24)	0.287
**Religion N(%)**				
Christian	566(70.84)	22.26(19.01, 25.89)	1	
Hindu	180(22.53)	32.22(25.75, 39.46)	1.66(1.15, 2.40)	0.007
Muslim	39(4.81)	23.08(12.10, 39.54)	1.05(0.48, 2.26)	0.906
Others and not stated	26 (3.21)	19.23(07.73, 40.37)	0.83(0.31, 2.25)	0.716
**Marital status N(%)**				
Married/co-habiting	292(36.41)	23.63(19.08, 28.87)	1	
Divorced/ legally separated	47(5.86)	21.28(11.57, 35.82)	0.87 (0.41, 1.85)	0.723
Never married	91(11.35)	19.78(12.72, 29.43)	0.80(0.45, 1.43)	0.445
Widowed	372(46.38)	26.88(22.60, 31.64)	1.19(0.83, 1.69)	0.340
**Education N(%)**				
None	48(6.09)	50.00(35.73, 64.27)	9.29(3.54, 24.33)	<0.001
Primary	560(71.07)	26.07(22.59, 29.88)	3.27(1.47, 7.30)	0.004
Secondary	108(13.71)	14.81(09.20, 22.97)	1.61(0.63, 4.15)	0.319
Tertiary/other	72(9.14)	09.72(04.62, 19.31)	1	
**Occupation N(%)**				
Associates /professionals / managers	192(24.12)	14.06(09.79, 19.79)	1	
Agriculture workers	113(14.20)	30.09(22.25, 39.30)	2.63(1.48, 4.66)	0.001
Unskilled / semi-skilled labourers	195(24.50)	24.62(19.03, 31.21)	2.00(1.18, 3.36)	0.009
Skilled labourers	131(16.46)	28.24(21.11, 36.67)	2.41(1.38, 4.20)	0.002
House wife	165(20.73)	30.30(23.71, 37.81)	2.66(1.57, 4.49)	<0.001
**Type of Accommodation N(%)**				
Detached house	763(94.78)	25.16(22.20, 28.37)	1	
Condominium/townhouse/ other	42(5.22)	14.29(06.33, 29.10)	0.50(0.21, 1.19)	0.118
**Tenure of Accommodation N(%)**				
Privately owned by spouse/ family	692(88.83)	26.01(22.87, 29.42)	1	
Rented from private landlord/NHA/housing/other	87(11.17)	18.39(11.47, 28.16)	0.64(0.36, 1.13)	0.125
**Living arrangements of chief respondent N (%)**			
Yourself	156(19.31)	16.03(11.01, 22.73)	1	
With Spouse	134(16.58)	19.40(13.50, 27.08)	1.26(0.69, 2.31)	0.452
With spouse and children	133(16.46)	24.81(18.13, 32.96)	1.73(0.97, 3.09)	0.065
With children alone	216(26.73	31.48(25.59, 38.03)	2.41(1.44, 4.03)	0.001
With other	169(20.92)	26.63(20.45, 33.87)	1.90(1.10, 3.29)	0.021
**Number of Comorbidities present N (%)**				
**None**	43(5.30)	13.95(06.19, 28.48)	1	
1	362(44.64)	19.34(15.57, 23.75)	1.48(0.60, 3.64)	0.395
2	205(25.28)	24.88(19.40, 31.31)	2.04(0.81, 5.12)	0.128
3	119(14.67)	28.57(21.08, 37.55)	2.47(0.95, 6.38)	0.062
≥4	82(10.11)	45.12(34.52, 56.18)	5.07(1.93, 13.32)	0.001
**Alcohol N(%)**				
Never	561(69.69)	26.56(23.06, 30.38)	1	
Less than once a month	158(19.63)	20.25(14.64, 27.32)	0.70(0.46, 1.08)	0.108
Less than once a week /at least Weekly	86(10.68)	15.12(08.89, 24.54)	0.49(0.27, 0.91)	0.025
**Smoking N(%)**				
Never	592(73.00)	25.00(21.67, 28.66)	1	
Ever	179(22.07)	22.35(16.79, 29.10)	0.86(0.58, 1.29)	0.469
Current	40(4.93)	25.00(13.63, 41.32)	1.00(0.48, 2.09)	1.000
**Self-reported Medical conditions N(%)**				
Arthritis	274(33.79)	27.01(22.05, 32.60)	1.23(0.88, 1.72)	0.220
Angina	8(01.00)	37.50(08.65, 79.17)	1.87(0.44, 7.90)	0.394
Diabetes	252(31.07)	31.75(26.26, 37.79)	1.74(1.24, 2.43)	0.001
Heart disease	84(10.36)	30.95(21.85, 41.82)	1.45 0.88, 2.37)	0.142
High cholesterol	104(12.82)	27.88(20.00, 37.42)	1.23(0.78, 1.95)	0.378
Hypertension /BP medicine	408(50.31)	25.49(21.48, 29.96)	1.12(0.82, 1.55)	0.473
Stroke	48(05.92)	58.33(43.57, 71.74)	4.88(2.68, 8.89)	<0.001
**Quality of life variables**			
**Impairment**				
**Eyesight**				
No	140(17.39)	17.86(12.30, 25.20)	1	
Yes	665(82.61)	25.86(22.67, 29.33)	1.60(1.01, 2.56)	0.047
**Hearing**				
No	322(40.00)	17.39(13.61, 21.95)	1	
Yes	483(60.00)	29.19(25.30, 33.42)	1.96(1.38, 2.78)	<0.001
**Climbing stairs**				
No	234(29.03)	11.54(08.01, 16.34)	1	
Yes	572(70.97)	29.72(26.11, 33.61)	3.24(2.09, 5.03)	<0.001
**Walking**				
No	243(30.19)	12.76(09.09, 17.61)	1	
Yes	562(69.81)	29.54(25.90, 33.46)	2.87(1.89, 4.36)	<0.001
**Ability to speak and understand**				
No	429(53.36)	14.92(11.84, 18.63)	1	
Yes	375(46.64)	35.20(30.51, 40.19)	3.10(2.20, 4.35)	<0.001
**IADL**				
0	50(6.17)	86.00(72.86, 93.36)	1	
1–2	72(8.88)	55.56(43.71, 66.80)	0.20(0.08, 0.51)	0.001
3–4	79(9.74)	37.97(27.79, 49.35)	0.10(0.40, 0.25)	<0.001
5–8	610(75.22)	13.93(11.40, 16.93)	0.03(0.01, 0.06)	<0.001
**Exercise N(%)**				
Moderate/ High	571(70.93)	14.36(11.71, 17.49)	1	
Low	183(22.73)	39.34(32.47, 46.67)	3.87(2.65, 5.64)	<0.001
None	51(6.34)	76.47(62.46, 86.39)	19.38(9.74, 38.56)	<0.001

**Figure 2:**
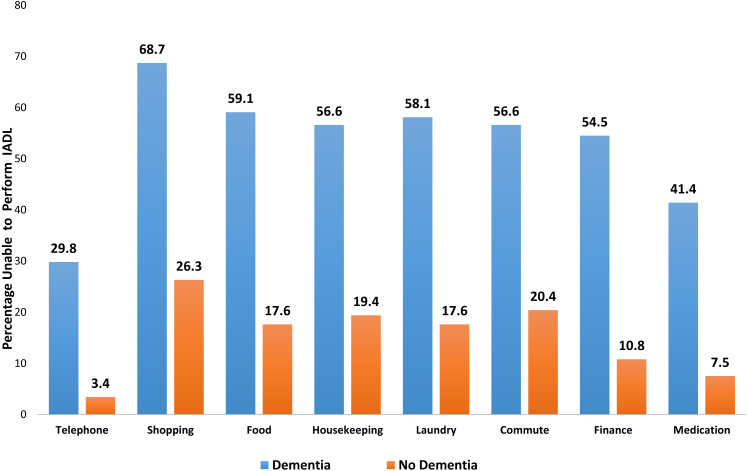
Bar chart showing difference in IADLs between those with dementia and those with no dementia.

**Figure 3:**
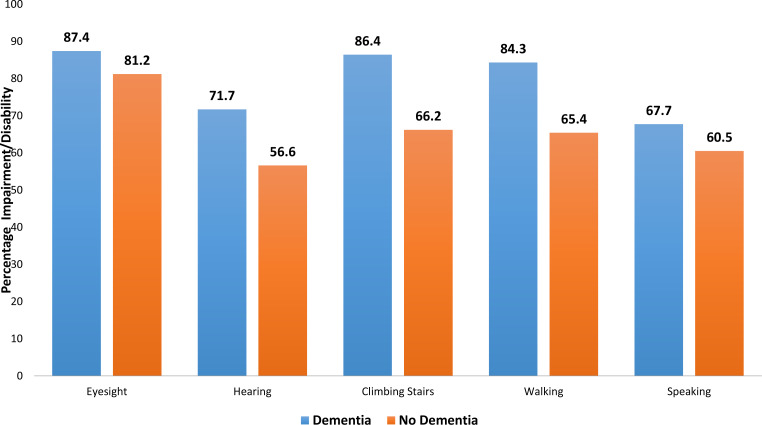
Bar chart showing differences in impairment/ disability between those with dementia and those with no dementia.

**Figure 4:**
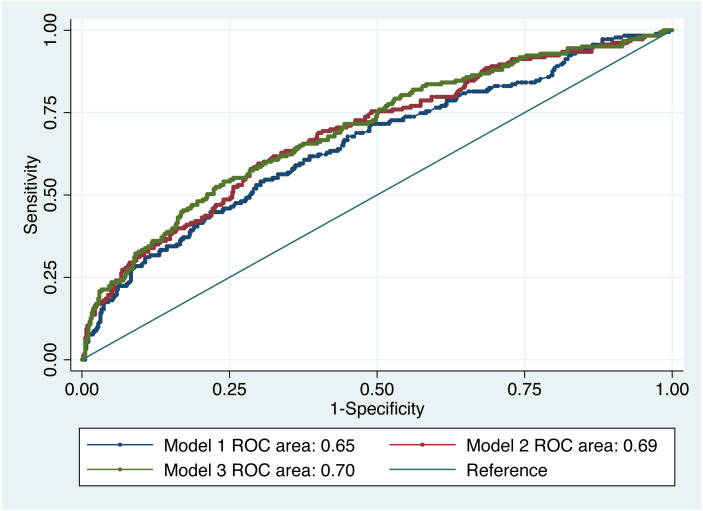
ROC curve of dementia risk prediction models for self-reported medical conditions based on multivariable logistic regression.

IADL and impairments were compared visually between people with/without dementia in Figs. 2 and 3. Table 2 shows the association between dementia and some of the self-reported medical conditions. With respect to the three developed models, there was no significant difference between them based on a test of difference between the AUROCs (Fig. 4). Model 1 adjusted for age, age^2^, gender, ethnicity and religion (AUROC = 0.65). Model II showed an improvement with the inclusion of education and occupation (AUROC = 0.69) which were also statistically significant. Model III showed only a slight improvement with the inclusion of marital status, type of accommodation, living arrangements, smoking and alcohol consumption (AUROC = 0.70). The GOF test and Hosmer and Lemeshow’s test were not significant for any of the models. Testing for equality between model I and II, there was significant difference (*p* = 0.01). The pairwise chi-square test for equality between model II and III showed that there was no significant difference. Since there was only a slight improvement with the inclusion of more factors in model III, we chose model II as the best fit model since it had a moderate number of non-modifiable variables. Independent associations were identified for diabetes and stroke in all models, but not for any other condition. Of the self-reported medical conditions, odds ratios for dementia were 4.93 (95% CI [2.64–9.23]) for stroke and 1.76 (95% CI [1.17–2.65]) for diabetes adjusting for age, age^2^, gender, ethnicity, religion, education and occupation.

**Figure 5:**
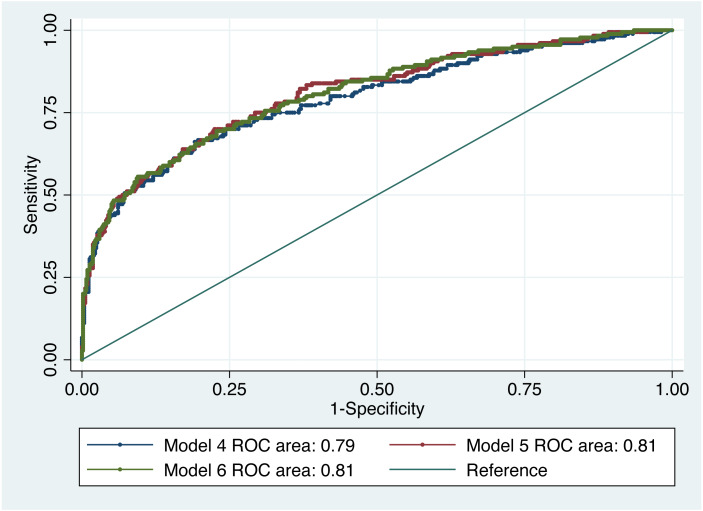
ROC curve of dementia risk prediction models for quality of life based on multivariable logistic regression.

Table 3 shows the association between dementia and IADL score, impairments and level of exercise. Three models were developed and Model V (AUROC = 0.81) was the best fit based on prediction accuracy with a minimum number of variables (Fig. 5). The GOF test and Hosmer and Lemeshow’s test were not significant for any of the models. There was no significant difference between model IV and model V, and model V and model VI. Being able to do IADLs had a protective effect. Compared to those who were not able to conduct any of the IADLs (0), those who were able to conduct only 1–2 IADLs had 77% lower odds (OR 0.23, 95% CI [0.07–0.75]), those who were able to conduct 3–4 IADLs were had 84% lower odds (OR 0.16, 95% CI [0.05–0.55]) and those who were able to conduct 5–8 IADLs were had 94% lower odds (OR 0.06, 95% CI [0.02–0.18]) to suffer from dementia. Compared to high to moderate level of exercise, those who did not exercise at all were more likely to have dementia (OR 6.95, 95% CI [2.02–23.90]) in model V.

## Discussion

The island of Trinidad is located off the east coast of Venezuela in the Caribbean sea, spanning an area of approximately 4,768 square km. It has a population of 1,328,019 with 48.7% males. It has a multi-ethnic multi-religious population with 35.4% of East Indian and 34.2% of African descent (Central Statistical Office (CSO), 2011). Our study found the prevalence of dementia to be 24.4% among the middle-old in Trinidad which is higher than that found in USA and Latin America (Prince et al., 2013). In the middle-old population in Trinidad having diabetes and stroke, low IADL score, and no exercise were more common in people with dementia.

**Table 2:** Multivariable models for association of self-reported medical conditions and dementia.

Covariates	Adjusted for age, age^2^, gender, ethnicity and religion (Model I)			Adjusted for age, age^2^, gender, ethnicity and , religion education, occupation (Model II)			Adjusted for age, age^2^, gender, ethnicity, religion, education, occupation, alcohol, , smoker, marital status, living arrangement of chief respondents and type of accommodation (Model III)		
**Self-reported Medical conditions**	OR (95% CI)	Robust SE	p>$ \left\vert z \right\vert $	OR (95% CI)	Robust SE	p>$ \left\vert z \right\vert $	OR (95% CI)	Robust SE	p>$ \left\vert z \right\vert $
Arthritis	1.19(0.86, 1.64)	0.197	0.299	1.07(0.76, 1.51)	0.186	0.709	1.03(0.72, 1.48)	0.189	0.875
Angina	1.13(0.29, 4.42)	0.786	0.859	1.07(0.26, 4.41)	0.773	0.927	1.39(0.40, 4.86)	0.888	0.607
Diabetes	1.79(1.23, 2.60)	0.342	0.002	1.76(1.17, 2.65)	0.367	0.007	1.81(1.19, 2.75)	0.387	0.006
Hypertension /BP medicine	0.85(0.59, 1.22)	0.156	0.382	0.83(0.57, 1.21)	0.158	0.330	0.79(0.54, 1.16)	0.154	0.231
Heart disease	1.30(0.79, 2.13)	0.328	0.307	1.24(0.76, 2.03)	0.311	0.392	1.33(0.82, 2.16)	0.330	0.245
High cholesterol	0.97(0.57, 1.69)	0.271	0.925	0.89(0.53, 1.51)	0.239	0.665	0.85(0.49, 1.46)	0.234	0.544
Stroke	5.25(2.75, 10.02)	1.730	<0.001	4.93(2.64, 9.23)	1.577	<0.001	4.40(2.30, 8.42)	1.456	<0.001
Number of observations	810			774			725		
Goodness-of-fit test (*p*-value)	0.62			0.41			0.19		
Hosmer-Lemeshow test (*p*-value)	0.19			0.25			0.39		
AUROC^*^	0.65 (0.61, 0.70)			0.69(0.64, 0.73)			0.70(0.65, 0.74)		

**Notes.**

* Null Hypothesis

area(model II) =area(model III) and *χ*^2^ = 5.99 , *p*-value = 0.05.

Reference Category: Arthritis: No; Angina: No; Diabetes: No; Hypertension /BP medicine: No; Heart disease: No; High cholesterol:No; Stroke: No.

### Univariable analyses

Age, gender, ethnicity and religion are non-modifiable risk factors for dementia and multiple morbidities. Our univariable analyses confirmed that women, those of East Indian descent and followers of Hindu faith were more likely to suffer from dementia. Among the middle-old population, dementia was associated with a lower level of education. The majority of individuals, about seventy five percent in the studied age range born between 1936 and 1945, had no educational qualifications. This picture of dementia in the elderly may change in the future reflecting mandatory schooling up to the secondary level in the younger generation in Trinidad. Education is a potentially modifiable risk factor that is believed to build cognitive reserves. Occupation was also a factor associated with dementia status. Being an agricultural worker, skilled labourer or housewife puts an individual at a higher risk for having dementia. Challenging jobs may decrease rates of cognitive decline and also build up an individual’s cognitive reserve. The findings of our study are consistent with reports that jobs that are both challenging and afford opportunities to take responsibility are associated with a reduced likelihood of dementia (Then et al., 2015; Qiu & Fratiglioni, 2018), although the explanation might also lie in better access to healthcare and the adoption of healthier lifestyles by those in higher educational and income categories. Individuals with more years of education might also be more likely to access more challenging jobs.

**Table 3:** Multivariable models for association of quality of life variables and dementia.

Covariates	Adjusted for age age^2^, gender, ethnicity, and religion (Model IV)			Adjusted for age, age^2^, gender, ethnicity, religion, education and occupation(Model V)			Adjusted for age, age^2^, gender, ethnicity education, religion, occupation, alcohol, smoker, marital status, living arrangement of chief respondents , and type of accommodation (Model VI)		
Quality of life variables	OR (95% CI)	Robust SE	p>$ \left\vert z \right\vert $	OR (95% CI)	Robust SE	p>$ \left\vert z \right\vert $	OR (95% CI)	Robust SE	p>$ \left\vert z \right\vert $
**Impairment**									
Eyesight	0.62(0.29, 1.32)	0.238	0.213	0.52(0.22, 1.20)	0.222	0.126	0.54(0.23, 1.22)		0.226	0.138	
Hearing	1.35(0.73, 2.53)	0.431	0.341	1.47(0.71, 3.06)	0.550	0.300	1.45(0.69, 3.06)	0.553	0.329
Climbing stairs	2.24(0.76, 6.65)	1.245	0.145	2.17(0.70, 6.73)	1.254	0.179	2.20(0.66, 7.35)	1.360	0.199
Walking	0.48(0.17, 1.37)	0.260	0.175	0.49(0.16, 1.48)	0.277	0.208	0.46(0.14, 1.46)	0.270	0.189
Ability to speak and understand	1.82(1.03, 3.23)	0.532	0.040	1.65(0.91, 2.99)	0.500	0.098	1.60(0.87, 2.96)	0.500	0.134
**IADL**									
1–2	0.26(0.08, 0.86)	0.160	0.028	0.23(0.07, 0.75)	0.139	0.015	0.18(0.05, 0.67)	0.120	0.010
3–4	0.17(0.05, 0.57)	0.105	0.004	0.16(0.05, 0.55)	0.101	0.004	0.14(0.04, 0.50)	0.090	0.003
5–8	0.07(0.03, 0.20)	0.038	<0.001	0.06(0.02, 0.18)	0.034	<0.001	0.05(0.02, 0.17)	0.031	<0.001
**Exercise N(%)**									
Low	1.96(1.16, 3.31)	0.524	0.012	1.83(1.07, 3.13)	0.502	0.028	1.94(1.12, 3.36)	0.543	0.018
None	5.53(1.92, 15.95)	2.989	0.002	6.95(2.02, 23.90)	4.380	0.002	6.64(1.94, 22.79)	4.178	0.003
Number of observations	798			763			719		
Goodness-of-fit test (*p*-value)	0.15			0.33			0.29		
Hosmer-Lemeshow test (*p*-value)	0.95			0.34			0.99		
AUROC *	0.79 (0.75, 0.83)			0.81 (0.77, 0.85)			0.81(0.77, 0.85)		

**Notes.**

* *H_0_: area(model IV) =area(model V) =area(model VI) and *χ*^2^ = 3.90, *p*-value 0.14.

Reference Category: Impairment “eyesight”: No; Impairment “Hearing”: No; Impairment “Climbing stairs”: No; Impairment “Walking”: No; Impairment “Ability to speak and understand”: No; IADL:0; Exercise: Moderate/ High.

Alcohol consumption compared to non-consumption was associated with lower odds of dementia, consistent with other research reporting that alcohol consumption is associated with a lower risk of any dementia or vascular dementia (Letenneur, 2004). Conversely, heavy alcohol binge drinking has been reported to be associated with increased risk of dementia (Järvenpää et al., 2005). In Trinidad and Tobago society, many consider it is a social norm when meeting with friends and family to have a few alcoholic beverages (Sinanan, 2017). The findings with respect to alcohol may, therefore, reflect the effect of greater social interaction. Those living with children alone were more likely to have dementia than those living alone by themselves, although this might reflect a higher level of dependency in the former group accounting for their living arrangements.

Those individuals having more than four co-morbidities were more likely to have dementia than those with no co-morbidities. Multimorbidity increases with age, as does dementia; such individuals are admitted to and stay longer in hospital with the attendant increase in complications, cost and mortality as well as increased use of health care services. Multimorbidity can reduce the quality of life of older people, and when dementia is a part of the condition, the situation may be even more demanding. Our univariable analyses showed that those with self-reported diabetes and stroke were more likely to suffer from dementia. This agrees with a study that found that multimorbidity is associated with increases in functional limitations, and the associations are stronger among women than among men and among adults aged 75 or older than among those aged 65 to 74 (Jindai et al., 2005). Age and gender will always play a role as will the duration of each co-morbidity because chronic diseases have time-varying effects.

Further, our univariable analyses revealed that impairment in eyesight, hearing, climbing stairs, walking and ability to speak and understand were more common in those having dementia. Individuals with dementia were more likely to be unable to perform all eight IADLs. Those who did not exercise at all and, by extension, lived sedentary lifestyles were also more likely to have dementia. Of the participants, 24% were assessed by the algorithm as having dementia and more than half of these individuals had impairment in IADLs. Shopping and commuting (Fig. 2) were among those activities that were adversely affected in participants with dementia. Studies have shown that there are differential effects of the type and frequency of social participation on IADL decline in older people (Tomioka, Kurumatani & Saeki, 2018) and that the type and frequency of social participation associated with IADL decline varies according to physical and mental functioning (Tomioka, Kurumatani & Saeki, 2018; Griffith et al., 2017).

### Multivariable analyses

We developed a series of multivariable models to identify the self-reported medical conditions and quality of life variables associated with dementia. Our multivariable analyses of self-reported medical conditions adjusting for age, age^2^, gender, ethnicity, religion, education and occupation (Model II) showed that those with self-reported diabetes and stroke were more likely to have dementia than those who did not have diabetes and stroke, respectively. This is not surprising in a country with high vascular risk, and is comparable to findings from another study (Chadee et al., 2013); however, it should be noted that their study population was limited to those who accessed a short-term financial assistance programme run by the Ministry of the People and Social Development in the Government of Trinidad and Tobago. The risk of dementia has been also associated with the duration and severity of diabetes (Livingston et al., 2020). Qiu & Fratiglioni (2018) suggest that interventions targeting multiple major vascular risk factors may represent a promising approach in reducing dementia risk and delaying its onset. It is also noteworthy that in our study only 14% of individuals with no comorbidity had dementia which was similar to that reported by another study (Browne et al., 2017).

Our multivariable analyses of quality-of-life variables adjusting for age, age^2^, gender, ethnicity, religion, education and occupation (Model V) revealed a persisting association of impairments with dementia. Qiu & Fratiglioni (2018) report that people who frequently engage in mentally stimulating activities (*e.g.*, learning, reading, or playing games) from young adulthood through midlife and old age are less likely to develop dementia. It is entirely possible that physical impairment impacted negatively on the ability of an individual to be active. Focusing on healthcare activities that may lead to reducing the likelihood of impairment such as eye care in diabetes and encouraging more activity would be beneficial in our middle-old population. Some studies have reported that aerobic exercises are associated with reduced risk of cognitive impairment and dementia, (Matura et al., 2017) and that physical activity not only improves fitness but protects brain cells (Ahlskog et al., 2011). However, patterns of physical activity change with age, generation, and morbidity and are different across sex, social class, and cultures. Meta-analyses of longitudinal observational studies of 1–21 years duration showed exercise to be associated with reduced risk of dementia (Livingston et al., 2020). The associations between leisure activity, not smoking, and increased survival still existed in those aged 75 years or more, with women living longer than men (Rizzuto et al., 2012). This is an important piece of information for caregivers and policymakers to take into consideration when planning care strategies.

### Strengths and limitations

Strengths of the study included its large size (allowing sufficiently precise estimates, as indicated by the widths of the confidence intervals) and household enumeration methodology to achieve a representative sample of the source population. A standard and widely used protocol was also used for dementia assessment and ascertainment. Considering potential limitations, this study was based upon face to face door-to-door interviews with individuals willing to participate. This may have introduced a selection bias at the participation stage, as well as through further missing data precluding dementia algorithm output (although the latter only accounted for 3% of participants lost). Although corroboration was sought from informants on exposures where possible, there may have been error arising from self-reporting bias, although this would more likely add error and obscure rather than exaggerate associations of interest. The data presented here were drawn from a larger study whose aim was focused on measuring the prevalence of dementia in Trinidad; thus not all measures of potential interest were available. Gender could also be treated as an effect modifier for dementia and multiple morbidities. The data also did not allow us to explore the effect of the environment on IADL, or dietary or other lifestyles as risk factors (Hendrie et al., 2001; Smith, Petot & Perry, 1999), or the relationship between APOE e4 and education or occupation. The survey did not collect information either on the duration of risk factors or the onset of dementia.

## Conclusion

Trinidad and Tobago, like most countries in the world, is facing the challenge of providing health care for its aging population. Accurate data on the health care status of the elderly as well as knowledge of factors that protect or put individuals at risk for dementia would provide the health care team with the information necessary for prudent allocation of limited resources to better serve this population. Further work is needed to determine if IADL evaluation has any role to play in the detection of dementia in at risk communities. In the context of the high levels of impairment and IADL, future studies should investigate how community and social capital could be used to improve care for our community dwellers with dementia. In the middle-old population, with high levels of dementia, the various types of impairment, levels of exercise and the inability of individuals to perform IADL (De Lepeleire et al., 2004), should be considered as a metric for implementing social/medical services needed for this risk group. Further studies may be conducted on prevalence of different types of dementia in Trinidad and Tobago.

##  Supplemental Information

10.7717/peerj.12040/supp-1 Supplemental Information 1 List of variables for dementia (75–84 yrs)

10.7717/peerj.12040/supp-2 Supplemental Information 2 STATA Codes Dementia

10.7717/peerj.12040/supp-3 Supplemental Information 3 The characteristics of the total middle-old population in the study Individuals that were considered to have dementia and those that did not are indicated. These groups were used for statistical analysis.

## Additional Information and Declarations

### Data Availability

The following information was supplied regarding data availability: The raw data and code are available in the Supplemental Files.
