# Supplementary material for: Health status risk factors and quality of life in 75–84-year-old individuals assessed for dementia using the short 10/66 dementia diagnostic schedule
Source: PeerJ. 2021 Aug 19;9:e12040. doi: 10.7717/peerj.12040 (PMC8380427; doi:10.7717/peerj.12040)
Supplement: Supplemental Information 1 [file peerj-09-12040-s001.docx]

| Study Variables (Description and Categories) | | |
| --- | --- | --- |
| Outcome Variable: Dementia, (0 = No; 1 = Yes) | | |
| Explanatory Variables | | |
| Age | Age of participants (Continuous variable) |  |
| Gender | Gender of participants (1=Male, 2=Female) |  |
| Ethnicity | Ethnicity of participants (1=African; 2=East Indian ; 3=Mixed African & East Indian; 4=Mixed Other/Others i.e. Caucasian/ Chinese/Indigenous/Portuguese/ Other ethnic groups) |  |
| Marital Status | Marital Status of participants (1=Divorced/Legally separated; 2=Married/ co-habiting; 3=Never married; 4=Widowed). |  |
| Education level | Education level of participants (1 = No Education; 2 = Primary; 3 = Secondary; 4 = Tertiary/other) |  |
| Religion | Religion of participants (1 = Christian; 2= Hindu; 3 = Muslim and Others) |  |
| Occupational level | Occupational level of participants (1 = Professional/Associate professional/Manager /Administrator/clerical worker/ secretary; 2=Agricultural worker; 3=Unskilled/Semi-skilled labourer; 4=skilled labourer; 5 =Housewife) |  |
| Type of accommodation | Establish type of accommodation of participants (1=Detached house and 2= Condominium/Townhouse/Other). |  |
| Tenure of accommodation | Establish type of tenure accommodation of participants (1=Privately owned by spouse/ family; 2=Rented from private landlord/NHA/housing /other). |  |
| Living arrangements of chief respondent | Do you live by (1=Yourself/alone ; 2= With Spouse; 3=With spouse and children; 4= With children alone; 5= With other) |  |
| Presence of Comorbidities | Presence of Comorbidity (1= No comorbidities; 2=one comorbidity; 3= two comorbidities; 3= three comorbidities & 4= more than or equal to four comorbidities). |  |
| Self-reported medical conditions in last 3 months | The following conditions in the last 3 month (Arthritis: 0=No & 1=Yes; Angina: 0=No & 1=Yes; Diabetic: 0=No & 1=Yes; Heart disease: 0=No & 1=Yes; High cholesterol: 0=No & 1=Yes; Hypertension /BP medicine: 0=No & 1=Yes; Stroke: 0=No & 1=Yes). |  |
| Alcohol | How often do you normally have a drink of something with alcohol in it?  (1 = Never; 2= Less than once a month and 3= Less than once a week /at least Weekly). |  |
| Smoking habit | 1 = Never; 2 = Ever and 3= current. |  |
| IADL score | 0=No Activity, 1= 1-2, 2=3-4 and 3=5-8 |  |
| Impairment level | 1=yes and 0=No |  |
| Exercise | Taking into account both work and leisure, would you say that you are physically active?  (1= Very much/fairly; 2= Not very much; 3= Not at all). |  |
